# Awake Surgery With Visual Pathway Mapping in Low Grade Glioma Surgery

**DOI:** 10.7759/cureus.22135

**Published:** 2022-02-11

**Authors:** Marcos V Sangrador-Deitos, Rodrigo Uribe-Pacheco, Juan C. Balcázar-Padrón, Sergio Díaz-Bello, Santiago Núñez-Velasco

**Affiliations:** 1 Neurosurgery, National Institute of Neurology and Neurosurgery "Manuel Velasco Suárez", Mexico City, MEX

**Keywords:** tractography, intraoperative stimulation, glioma resection, visual pathway, awake surgery

## Abstract

The visual pathway and its defects have been thoroughly studied in clinical correlation to temporal lobe lesions related to epilepsy and traumatic lesions. Nevertheless, its clinical correlation and other decision-making have not been addressed regarding neoplastic lesions. We present a case report of a 28-year-old man with a one-year history of generalized seizures and left superior homonymous quadrantanopia, with no other neurological disturbance on physical examination. According to diffusion tensor imaging tractography, MRI demonstrated a non-enhancing, right temporal lesion disrupting the visual pathway. An awake surgery with direct cortical electrostimulation of visual pathways was performed with subtotal resection of the tumor to preserve visual function, confirmed with postoperative MRI. Histopathological studies revealed a fibrillary astrocytoma.

Surgical technique aided with intraoperative cortical and subcortical stimulation involving low-grade gliomas in eloquent areas is an exceptionally suitable procedure for complex cases where the visual pathway is compromised. Our objective is to describe how intraoperative mapping of visual function is performed in our institution and to comment on the relevant technical nuances, which can serve as a practical guideline for young neurosurgeons, as no previous cases have been reported in our country.

## Introduction

In 1907, Dr. Adolf Meyer, a Swiss psychiatrist, first described “the peculiar detour of the ventral portion of the geniculocalcarine pathway.” He concluded that a portion of the optic radiation, after leaving the geniculate body, plunged anteriorly into the temporal lobe through the tip of the horn of the ventricle before turning back towards the calcarine cortex [1]. Dr. Harvey Cushing recalls, in his paper about field defects after temporal lobe lesions, about a patient with epilepsy resulting from a gunshot injury, in which the bullet was lodged in the right temporal lobe and was diagnosed with no visual field defect. However, in 1910, Dr. Meyer visited the ward and suggested the visual field to be divided into <30° intervals to detect the visual deficit, and indeed, he was right [2]. Since then, a lot has been studied about visual defects following temporal lobe epilepsy surgery that produce “markedly incongruous superior quadrantanopia” [3], but not much concerning the field of neoplastic lesions.

Gliomas are the most common primary malignant brain tumor among adults, with an incidence reported from 4.67 to 5.73 per 100,000 people [4]. The World Health Organization, in its latest classification system, establishes four categories from grade I to IV depending on histopathologic features such as atypia, anaplasia, mitotic activity, and necrosis [5]. Low-grade gliomas include grade I tumors, which lack any prior features, and grade II tumors characterized only by atypia. Lesions included in this category encompass diffuse astrocytoma, pilomyxoid astrocytoma, pleomorphic xanthoastrocytoma, oligodendrogliomas (WHO grade II), and subependymal giant cell astrocytoma (SEGA), typical from tuberous sclerosis; pilocytic astrocytoma, ganglioglioma, dysembryoplastic neuroepithelial tumor, among others [6]. These tumors are most common in young adults from the second to the fourth decades of life, being the clinical signs and symptoms attributed to the mass effect. Seizures are the debuting symptom in up to 80% of cases [7].

## Case presentation

**Figure 1:**
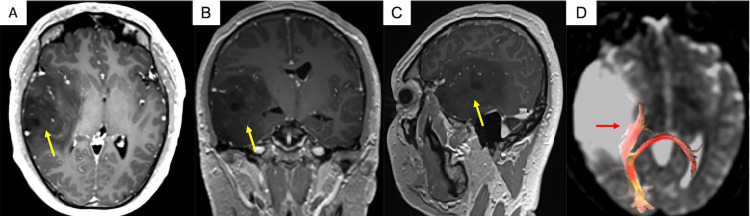
Contrast-enhanced MRI and DTI tractography. Contrast-enhanced MRI: (A) axial, (B) coronal, and (C) sagittal reconstructions in which a homogeneous, non-enhancing right temporal lesion is observed (yellow arrows). (D) DTI tractography showing displacement of the right visual pathways (red arrow). DTI, diffusion tensor imaging

**Figure 2:**
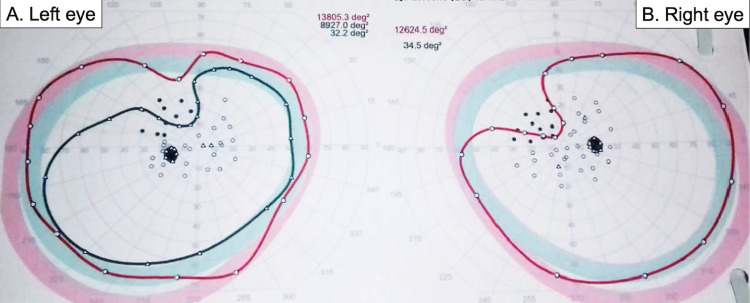
Preoperative visual field campimetry. Octopus visual field with bilateral 20/20 visual acuity and 8/8 Ishihara. (A) Left eye: temporal superior quadrantanopia with I4e and III4e stimuli, or relative quadrantanopia. (B) Right eye: nasal superior quadrantanopia with III4e stimuli.

A 28-year-old man presented with a one-year history of generalized seizures to our institution; at admission, the general and neurological evaluation revealed no disturbances. Contrast-enhanced MRI demonstrated a non-enhancing, right temporal lesion, measuring 52 mm x 50 mm x 56 mm, and diffusion tensor imaging (DTI) tractography showed tumoral visual pathway invasion, affecting the right parietal portion, which carries information from the inferior quadrant of both retinas (Figure 1A-D). Neuro-ophthalmologic preoperative evaluation demonstrated a left superior homonymous quadrantanopia, which can be explained by the disruption of Meyer´s loop (Figure 2).

**Figure 3:**
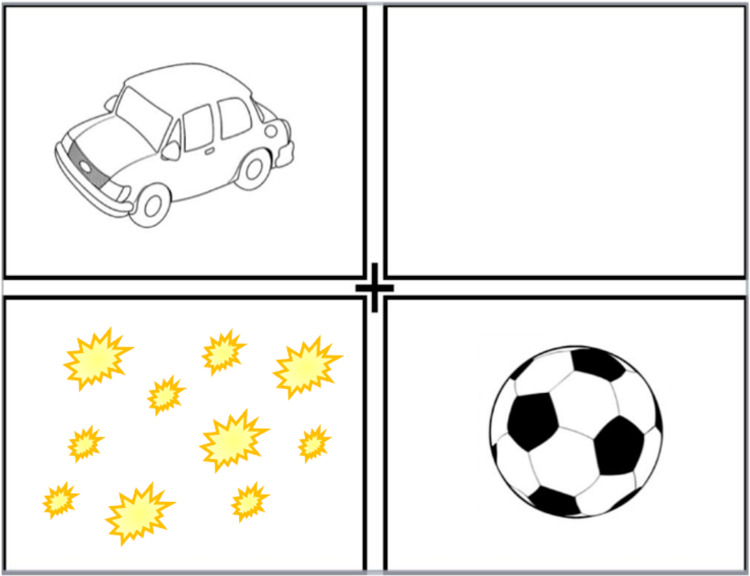
Visual pathway evaluation chart. Whenever the parietal visual pathway was stimulated, the patient referred as “flashlights under the car.”

The situation was thoroughly discussed with the patient and his relatives, as complete resection of the tumor represented a high risk of an added visual field defect, in this case, a complete homonymous right hemianopia. Due to the situation's complexity and the consequences, it could bring to the patient's quality of life, the decision to respect functional areas was taken. We decided to perform an awake surgery with direct cortical electrostimulation of visual pathways. The patient was instructed to inform us whenever he detected an alteration in his visual field using an evaluation chart (Figure 3).

**Figure 4:**
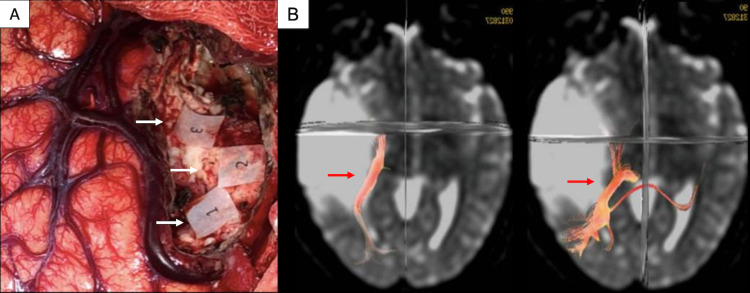
Intraoperative visual pathways mapping and DTI tractography. (A) Intraoperative photography showing visual pathways mapping after direct cortical electrostimulation (white arrows). (B) DTI tractography showing displacement and invasion of the optic radiations (red arrows). DTI, diffusion tensor imaging

A conventional pterional approach and a three-phase asleep-awake-asleep anesthetic technique for intraoperative mapping were performed. Total intravenous general anesthesia (TIVA) with fentanyl, propofol, dexmedetomidine, and a single dose of rocuronium, was used for induction. A laryngeal mask was placed for airway management. Subsequently, anesthetic maintenance was performed with propofol, fentanyl, and dexmedetomidine in the first asleep phase. An intrinsic lesion affecting the middle and inferior temporal gyri was found, with a rostral displacement of the superior temporal gyrus. The vein of Labbé was used as the caudal limit for the resection, which was medially directed, and the patient was awakened. The patient's emersion was initiated through metabolic lysis of the drugs (fentanyl and propofol), spontaneous ventilation, aspiration of secretions, removal of the laryngeal mask, and support ventilation with nasal cannulas. During visual tests, anesthesia maintenance was carried out with low-dose dexmedetomidine in the second awake phase. We mapped the optic radiations all the way to the lateral wall of the temporal horn of the lateral ventricle, preserving these functional locations (Figure 4A,B). We used a bipolar electrode that delivers a biphasic current (Ojemman cortical stimulator, Integra Lifesciences Services, Saint-Priest, France) to the brain of the patient. The current intensity was progressively increased, using 1-mA amplitude increments (from a baseline of 1-mA) until a final amplitude of 5-mA, in which a functional response was elicited. Whenever the optic radiations were stimulated, the patient was referred to as seeing “flashlights under the car” in the visual pathway evaluation chart. Eloquent areas were properly identified using marked paper tags. The expected result of cortical stimulation in this area was visual stimuli on the left inferior quadrants. The induction and maintenance of the third asleep phase were performed with the same drugs as for the first phase, and airway management was achieved by using a Fastrack laryngeal mask. Conventional closure was performed, and the surgery was concluded with no eventualities.

**Figure 5:**
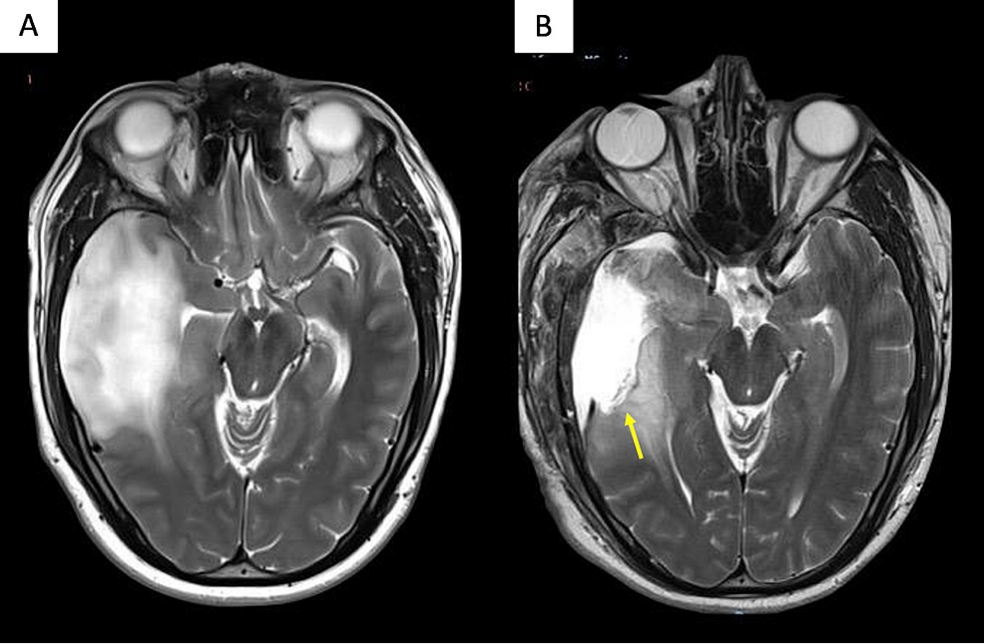
Preoperative and postoperative T2 sequence MRI. (A) Preoperative and (B) Postoperative T2 sequence MRI showing residual tumor on the most caudal and medial portion of the lesion (yellow arrow), which exactly corresponds with the optic radiations pathway and marks the limit of our cortical mapping.

**Figure 6:**
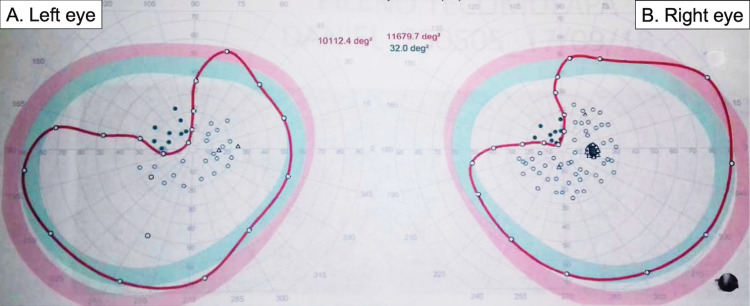
Postoperative visual field campimetry. Octopus visual field with bilateral 20/20 visual acuity and 8/8 Ishihara. (A) Left eye: temporal superior quadrantanopia with III4e stimuli. Left superior homonymous quadrantanopia. (B) Right eye: nasal superior quadrantanopia with III4e stimuli.

The patient´s postoperative course was uneventful. Postoperative MRI was performed, and it evidenced a subtotal resection with minimal residual tumor, which was left behind to spare visual function (Figure 5). Although a total resection would have been desired, the patient´s will and priority was to preserve his visual function under an acceptable range, which was achieved, as shown in the postoperative visual field campimetry, where a left superior homonymous quadrantanopia was found, the same as the preoperative findings (Figure 6). The histopathological report revealed a low-grade neoplastic lesion morphologically compatible with a fibrillary astrocytoma (WHO II diffuse glioma).

## Discussion

Early surgery is currently the treatment of choice when facing a patient with low-grade glioma. The extent of resection (EOR) has been reported as one of the most significant predictors of overall survival [8]. Smith et al. demonstrated that among 216 patients, those treated with an EOR of at least 90% had 5- and 8-year overall survival rates of 97% and 91%, respectively, whereas those who received an EOR of less than 90% had 5- and 8-year overall survival rates of 76% and 60%, respectively [9]. However, when trying to achieve a more considerable EOR, functionality, and quality of life sometimes are sacrificed because of damage to eloquent areas.

Consequently, in the past two decades, awake patient mapping surgery has shown to be a valuable tool to optimize resection rates in eloquent areas and minimize neurological deficit while improving postoperative functionality and quality of life [10]. As in our case, awake surgery allows not only the mapping of cortical structures but also subcortical white matter tracts [11]. The majority of awake surgery reports have focused on language areas to avoid permanent oral or written deficits [12]. Nonetheless, there have also been multiple reports of intraoperative nonlanguage mapping involving motor, sensory, vestibular, memory, and calculation areas, thus allowing a more functional outcome [13]. It was not until 2004 that Duffau et al. reported the first case of subcortical mapping of the visual pathways using intraoperative electrical stimulations in a 26-year-old female with a nine-month seizure history who had a tumor invading the right temporal lobe and the temporo-occipital junction. The patient had no motor or language deficit during the postoperative course, but ophthalmologic examination reported a left superior quadrantanopia with normal visual acuity. The histopathological examination reported a low-grade glioma (WHO II) [14]. It is not rare to obtain this kind of result; in 2012, Gras et al. reported a series of 14 patients in which subcortical electrical mapping of optic radiations was used, resulting in one patient with homonymous hemianopia and twelve with quadrantanopia [15].

Visual field defects account for necessary functionality and quality of life outcomes in the postoperative course. Defects such as quadrantanopia may be acceptable, as it is usually asymptomatic, and in most countries, it is not a contraindication for driving [16]. On the other hand, homonymous hemianopias have been shown to impact the patients´ quality of life severely [17]. The five main concerns are the desire for work, driving motor vehicles, independence, socializing, and freedom from drugs [18]. Functional outcomes, including neurocognitive and emotional components, must always be taken into account when trying to achieve the maximal EOR, as they could be predictors of survival, as shown in a Finnish study in which depressed patients with low-grade gliomas had a significantly shorter survival time, 3.3-5.8 years, compared to non-depressed patients, 10.0-11.7 years [19].

## Conclusions

Functional neuro-oncology, preserving functions and quality of life, has become an essential aspect of glioma surgery. Although the EOR is well known to be one of the most important factors for overall survival, as a surgeon, one must outweigh a reduction in progression-free and overall survival versus a completely functional patient. Functional neuro-oncology proves the essential role of using awake surgery techniques aided with intraoperative cortical and subcortical stimulation in low-grade gliomas involving eloquent areas.
